# Supplementary material for: Co-Crystallization Approach to Enhance the Stability of Moisture-Sensitive Drugs
Source: Pharmaceutics. 2023 Jan 5;15(1):189. doi: 10.3390/pharmaceutics15010189 (PMC9864382; doi:10.3390/pharmaceutics15010189)
Supplement: Supplementary file 1 [file pharmaceutics-15-00189-s001.zip › pharmaceutics-2032571-supplementary.pdf]

*pharmaceutics*

# Supplementary Materials: Co-crystallization approach to enhance the stability of moisture-sensitive drugs

**Madhukiran R. Dhondale, Pradip Thakor, Amritha G. Nambiar, Maan Singh, Ashish K. Agrawal, Nalini R. Shastri and Dinesh Kumar**

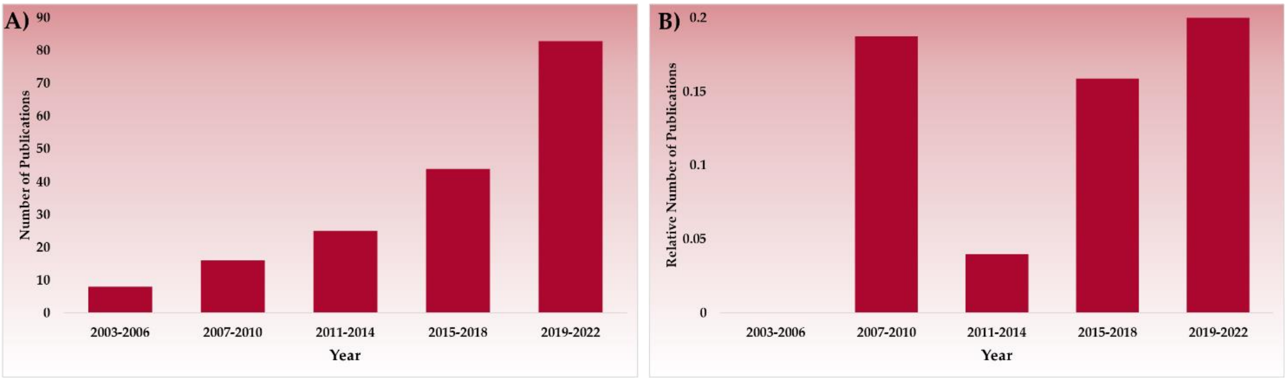


**Figure S1.** Trend of scientific publications from 2003 to 2022 for **A)** Drug Stability, Hygroscopicity, & **B)** Co-crystals, Drug stability and Hygroscopicity. Data taken from Web of Science.

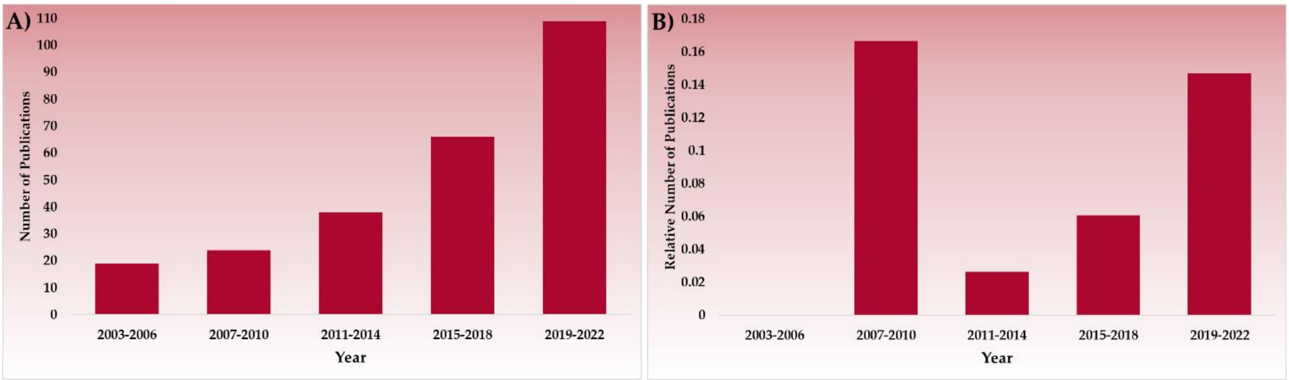


**Figure S2.** Trend of scientific publications from 2003 to 2022 for **A)** Drug Stability, Hygroscopicity, & **B)** Co-crystals, Drug stability, Hygroscopicity. Data taken from Scopus.
